# The Swara-Informed model of autonomic synchrony in psychotherapy: theoretical foundations and hypotheses

**DOI:** 10.3389/fpsyg.2026.1853837

**Published:** 2026-08-24

**Authors:** Aarsh Ojas Parasar Pandey

**Affiliations:** Department of Psychology, Indira Gandhi National Tribal University, Amarkantak, India

**Keywords:** autonomic regulation, autonomic synchrony, consciousness, pranayama, psychotherapy, Swara yoga, unilateral nostril breathing, window of tolerance

## Abstract

**Background:**

Autonomic dysregulation is increasingly recognized as a central mechanism complicating psychotherapeutic work across anxiety, trauma-related, and mood disorders. Persistent hyperarousal (sympathetic activation) or hypoarousal (persistent parasympathetic activation or shutdown/ dissociation) may put clients out of their “window of tolerance,” thus limiting access to cognitive, emotional, and interpersonal processes that are essential to the effectiveness of psychotherapy. Swara yoga, a traditional Indian system, assigns functional significance to nasal dominance and unilateral nostril breathing (UNB). According to this system, right nostril breathing is said to be activating, while left nostril breathing is calming. Contemporary psychophysiological studies on UNB and related breathing techniques have shown that nostril-specific breathing can alter autonomic indicators, such as heart rate variability. In contrast, slow-paced breathing tends to increase vagal tone and enhance autonomic flexibility.

**Objective:**

This conceptual analysis integrates Swara yoga's principles with current knowledge of autonomic physiology to propose a “Swara-informed autonomic synchrony model” for psychotherapy. The proposed model aims to provide state-contingent use of unilateral nostril breathing as an adjunct to evidence-based psychological interventions.

**Methods:**

Based on empirical investigations of UNB, alternate nostril breathing, heart rate variability, and trauma-related autonomic dysregulation, we developed a state-based clinical framework. Rather than assigning a fixed autonomic profile to each diagnostic category, we distinguished two broad psychophysiological states: hyperarousal (autonomic surge) and hypoarousal (shutdown), and mapped these states to nostril selection based upon Swara yoga.

**Model:**

We propose that slow left nostril breathing should be used as a parasympathetic biasing, down-regulatory adjunct to psychotherapy when the client presents with hyperarousal-dominant states such as panic, hyperarousal-dominant PTSD, or agitated anxiety. When hypoarousal-dominant states such as psychomotor-retarded depression, dissociative PTSD, and negative symptom presentations are evident, we propose that slow right nostril breathing should be used as a sympathetic biasing, up-regulatory adjunct to psychotherapy. In both cases, UNB would be used as a short-term, inexpensive preparatory regulatory technique embedded in standard psychotherapeutic procedures (e.g., exposure, behavioral activation, trauma processing).

**Conclusion:**

The Swara-informed autonomic matching model provides a culturally grounded framework that hypothesizes how nostril-specific breathing may be integrated into psychotherapy and provides a basis for future empirical investigation. We formulated testable hypotheses and study designs to investigate whether state-matched UNB yields greater benefits than slow-paced breathing. Finally, we discuss several potential limitations, safety issues, and ethical considerations relevant to this research area.

## 1 Introduction

Autonomic dysregulation is increasingly recognized as a transdiagnostic mechanism that complicates psychotherapeutic work across a range of clinical presentations, including individuals suffering from anxiety disorders, trauma-related disorders, and mood disorders (U.S Department of Veterans Affairs, 2013; Beutler et al., 2022). This recognition has provided the opportunity to develop new therapeutic adjunctive strategies targeting autonomic regulation to enhance therapeutic outcomes. Breathing-based interventions are one type of adjunctive strategy that has demonstrated some success in reducing symptoms of hyperarousal and hypoarousal (Beutler et al., 2022) and, consequently, improving the effectiveness of psychotherapies (Beutler et al., 2022). Persistent states of hyperarousal are characterized by sympathetic nervous system dominance, with elevated physiological activation, increased vigilance for potential threats, and increased physiological mobilization (Beutler et al., 2022). Hypoarousal represents a state of shutdown or parasympathetic dominance characterized by decreased physiological activity, dissociative experiences, and reduced behavioral readiness (Beutler et al., 2022). Such shifts in arousal experiences can push clients outside their “window of tolerance,” a construct that describes the optimal zone of autonomic arousal for effective cognitive, emotional, and interpersonal processing. Outside this window, a hyperarousal state limits reflective capacity through excessive threat appraisal and avoidance behaviors (Beutler et al., 2022), whereas a hypoarousal state impairs engagement through emotional numbing and motivational deficits (Beutler et al., 2022).

Breathing-based interventions have emerged as a promising cost-effective adjunctive strategy to psychotherapy. Evidence suggests that these practices can successfully modulate autonomic responses, such as heart rate variability (HRV), reduce subjective distress, and improve treatment engagement (Yadav et al., 2016; Steffen et al., 2021). Additionally, these techniques have been incorporated into various forms of cognitive behavioral therapy (CBT), trauma-focused treatments, and emotion regulation protocols, and have often shown benefits for both physiological and psychological outcomes (Steffen et al., 2021). However, most

current approaches utilize generic paced breathing. There are currently no culturally grounded nostril-specific practices that may target differential autonomic biasing.

Swara Yoga, a traditional Indian system that assigns functional significance to nasal dominance and unilateral nostril breathing (UNB), claims that right nostril breathing (Surya Nadi) is associated with activating and mobilizing, while left nostril breathing (Chandra Nadi) is associated with calming and restorative processes (Shiva Swarodaya (trans.), 1984). Some preliminary psychophysiological research provides some support for the notion of nostril-specific effects on HRV and cardiovascular parameters, suggesting that UNB may differentially influence sympathetic and parasympathetic activity (Pal et al., 2014; SAS Publishers, 2015). Despite growing empirical support, the systematic integration of autonomic regulation into mainstream psychotherapeutic practice remains limited, even though state-contingent autonomic regulation is a critical clinical need.

Despite emerging evidence that both unilateral nostril breathing and slow-paced breathing can modulate autonomic markers and subjective distress, there is currently no coherent framework for their systematic integration into mainstream psychotherapy. Existing breathing protocols are typically applied in a generic (slow-paced), non-state-contingent manner, with little guidance on matching specific techniques to hyperarousal versus hypoarousal presentations or to particular therapy tasks. This conceptual gap limits both clinical implementation and the design of mechanistically informed trials that could test nostril-specific and state-matching effects against active controls.

This article proposes a novel Swara-informed model of autonomic synchrony for psychotherapy. Rather than assigning fixed autonomic profiles to diagnostic categories, which risks oversimplifying the heterogeneity of clinical presentations, we recommend a state-based approach. Slow left nostril breathing should be used to down-regulate hyperarousal states, and slow right nostril breathing should be used to up-regulate hypoarousal states as brief adjuncts to evidence-based psychological treatments. This model incorporates Swara yoga principles with modern autonomic science, providing a culturally resonant, mechanistically oriented framework for clinical and health settings. The novelty of this article lies not in demonstrating the efficacy of unilateral nostril breathing, but in proposing a theoretically integrated framework that combines Swara Yoga with contemporary models of autonomic regulation to generate testable clinical hypotheses.

In this article, we use the term *consciousness* in a pragmatic, clinical sense to refer to the organization and quality of awareness, how present, integrated, and responsive a person is to internal and external experience, rather than to metaphysical questions about the nature of mind. We use the term *autonomic synchrony* to describe the alignment between Swara patterns (nostril-specific breath flow) and autonomic nervous system state, such that breath, physiological arousal, and momentary conscious experience operate as a coupled system during psychotherapy rather than independently.

In this article, we first review the literature on autonomic dysregulation in psychotherapy. Then, we examine empirical evidence from studies on UNB and related breathing practices. We then describe the core Swara Yoga principles relevant to clinical application. Next, we propose the autonomic synchrony model, including clinical decision-making and integration strategies. Finally, we formulate five testable hypotheses and outline an empirical research agenda to evaluate the model.

## 2 Autonomic regulation, trauma, and clinical health psychology

### 2.1 Hyperarousal and hypoarousal states in clinical disorders

Many clinical presentations are characterized by distinct autonomic profiles that influence therapeutic processes and outcomes. Hyperarousal states, characterized by sympathetic dominance, increased heart rate, decreased HRV, and symptoms such as hypervigilance, exaggerated startle response, irritability, and sleep disturbances, are prevalent in many anxiety disorders and the arousal/reactivity cluster of post-traumatic stress disorder (PTSD) (U.S Department of Veterans Affairs, 2013; Beutler et al., 2022). For example, DSM-5 PTSD criteria include several symptoms indicative of sustained mobilization, such as reckless behavior, hypervigilance, exaggerated startle behavior, difficulty concentrating, and sleep problems, all of which can impair exposure tolerance and cognitive reappraisal (U.S Department of Veterans Affairs, 2013).

On the other hand, hypoarousal states are associated with dorsal vagal dominance, decreased sympathetic outflow, and symptoms such as psychomotor retardation, emotional numbing, social withdrawal, and motivational deficits (Beutler et al., 2022). Symptoms consistent with hypoarousal are common in melancholic depression, negative-symptom schizophrenia spectrum disorders, and dissociative presentations (Beutler et al., 2022). The DSM-5 dissociative subtype of PTSD, characterized by depersonalization and derealization in addition to core PTSD symptoms, exemplifies predominantly hypoarousal-dominant trauma states in which shutdown and detachment limit the client's ability to engage in trauma processing or behavioral activation (U.S Department of Veterans Affairs, 2018; Beutler et al., 2022).

It is critical to note that these states are not permanently assigned to any diagnosis; rather, they vary within individuals and illness phases. Thus, therapists must utilize flexible, state-responsive regulation to effectively address the varied autonomic states (Beutler et al., 2022).

### 2.2 The window of tolerance and therapeutic process

The “window of tolerance” framework describes the ideal range of autonomic arousal for effective therapeutic engagement (Siegel, 1999). Hyperarousal (sympathetic dominance) leads to fight-flight responses, threat-focused attention, physiological mobilization, cognitive inflexibility, and avoidance, whereas hypoarousal (dorsal vagal shutdown) results in emotional numbing, psychomotor retardation, dissociation, and motivational deficits (Ogden et al., 2006; Beutler et al., 2022).

Exposure-based therapies encounter dropout rates of 20-40% in hyperarousal clients because of intolerable anxiety surges (Foa et al., 2005; Lanius et al., 2010). Conversely, hypoarousal-dominant PTSD (dissociative subtype) clients disengage due to emotional detachment (Lanius et al., 2010). Cognitive-behavioral therapy (CBT) for anxiety encounters either perseverative threat cognition (hyperarousal) or homework non-compliance (hypoarousal depression) (Hofmann and Smits, 2008). Q15 activation fails when hypoarousal state prevents task initiation (Dimidjian et al., 2011). Breathing-based interventions target vagal tone and HRV, which have been shown to correlate with emotional regulation and treatment adherence (Steffen et al., 2021). Higher baseline values predict improved regulation/alliance, and session improvements have been correlated with outcomes (Geisler et al., 2010; Steffen et al., 2021). Breathing-based interventions enhance vagal pathways via respiratory sinus arrhythmia and reliably increase HFnu during practice (Gerritsen and Band, 2018).

However, generic approaches often fail to adequately address the complexity and diversity of autonomic states (Chin et al., 2024), highlighting the need for culturally sensitive, mechanistically grounded models such as the one proposed here. Thus, the Swara-informed autonomic synchrony model addresses these gaps by employing state-contingent nostril selection (down-regulatory effect of left *Ida*, mobilizational effect of right *Pingala*) as psychotherapy adjuncts. Unlike uniformly paced breathing, Swara hypothesizes a differential autonomic biasing that can be tested against generic controls. Additionally, preliminary HRV support exists for this model, while honoring traditional rhythm analysis (Pal et al., 2014; Shiva Swarodaya (trans.), 1984).

## 3 Empirical foundations for one-nose breathing

### 3.1 One-nose breathing and heart rate variability (HRV)

HRV analysis using frequency-domain metrics offers an objective assessment of autonomic function. Low-frequency normalized units (LFnu) reflect combined sympathetic and parasympathetic activity (0.04–0.15 Hz), high-frequency normalized units (HFnu) primarily indicate parasympathetic and vagal tone via respiratory sinus arrhythmia (0.15–0.40 Hz), and the LF/HF ratio estimates sympathovagal balance (Task Force of the European Society of Cardiology and the North American Society of Pacing and Electrophysiology; Berntson et al., 1997).

Several studies have demonstrated that UNB has differential effects on HRV markers. In a seminal study, Pal et al. (2014) used a 6-week randomized controlled trial (RCT) with 90 young adults, randomly assigned to practice slow breathing (−6 breaths/min) for 30 min daily through the right, left, or alternate nostrils. Participants practicing right-nasal breathing showed a significant increase in LFnu (+ 15.2%) and LF/HF (+ 22.4%) and decreases in HFnu (−18.7%), indicative of enhanced sympathetic influence and decreased parasympathetic tone. Conversely, left-nasal breathing resulted in opposite patterns: HFnu (+ 21.3%), LFnu (−12.8%), and LF/HF (−19.6%), suggesting enhanced vagal tone and improved sympathovagal balance. Additionally, cardiovascular outcomes were similarly aligned: right-nasal practice increased systolic and diastolic blood pressure (+ 4.2/+ 3.1 mmHg), whereas left-nasal practice decreased them (−5.6/−3.8 mmHg), suggesting potentially clinically meaningful autonomic changes (Pal et al., 2014).

These results also extend to clinical populations. Telles et al. (2024) reported that nostril-regulated yoga breathing improved HRV time-domain measures (RMSSD + 14.3%) across protocols. Although the effects varied based on baseline autonomic state and breathing pattern, they highlighted individual differences. Block et al. (1989) demonstrated similar cognitive-performance relationships. Right nostril breathing has been associated with improved verbal task performance, and left nostril breathing with enhanced spatial performance, consistent with proposed hemispheric lateralisation effects; however, findings remain mixed and context-dependent (Telles et al., 2024; Block et al., 1989).

Given that these brief (5–15 min) protocols align with the timing of psychotherapy sessions, these results position UNB as a feasible adjunct to existing therapies rather than a separate intervention (Steffen et al., 2021).

### 3.2 Alternate-nostril and paced-breathing as context

Alternate-nostril breathing (ANB) and slow-paced breathing provide important context for understanding UNB effects, as both methods have been shown to enhance parasympathetic function across diverse populations. Yadav et al. (2016) randomly assigned healthy adult volunteers (*n* = 50) to either 15 min of ANB or slow breathing, and found that ANB significantly increased high-frequency power (HF: + 28.4%, *p* < 0.01) and the Root Mean Square Successive Differences (RMSSD: + 22.1%), and reduced sympathetic markers (LF/HF Ratio: −18.3%, *p* < 0.05).

Similarly, clinical data strengthen the generalisability of these results. Zhao et al. (2025) conducted an RCT in patients with chronic heart failure (*n* = 120). They found that slow-paced breathing (6 breaths/min, 20 min/day x 12 weeks) was associated with improved HRV [RMSSD +34.2% (*p* < 0.001), HFnu + 19.7%]and psychological distress (HADS Anxiety −4.2), with slow-paced breathing outperforming wait-list controls. Similarly, Pozzato et al. (2025) examined slow-paced breathing in individuals following traumatic injury (*n* = 85). They found that a 5-min pacing reduced state anxiety [−15.6% (*p* < 0.01)] and increased HFnu (12.4%), with effects mediated by respiratory sinus arrhythmia.

A meta-analysis of 15 RCTs confirmed that slow-paced breathing resulted in moderate reductions in anxiety (*p* < 0.001) compared with passive controls (Chin et al., 2024). Nonetheless, nostril dominance may produce directional biases. ANB studies suggest additional effects of nostril dominance beyond breathing rate alone, thereby justifying UNB-specific dismantling (Yadav et al., 2016; Telles et al., 2024).

### 3.3 Psychological outcomes and proposed mechanisms

In addition to its physiological effects, UNB produces promising psychological effects. Vanutelli et al. (2024) reported that both nostril practices reduced stress and mind-wandering, with differences between nostril practices suggesting that right-nasal breathing improved vigilance and left-nasal breathing improved relaxation, providing evidence of nostril-specific cognitive modulation.

Additionally, cognitive-performance studies support these findings. Block et al. (1989) found that left-nostril breathing improved verbal fluency (right-hemisphere tasks), while right-nasal breathing improved spatial rotation (left-hemisphere tasks), supporting Swara's hemispheric claims (Werntz et al., 1987). Clinical analogs: Telles et al. (2013) reported that ANB improved attention in hypertensive subjects (Stroop Test), supporting potential therapeutic application.

While the effects of nostrils are promising, they are probabilistic and protocol-dependent, supporting their utility as targeted adjuncts, but not as standalone interventions.

Unlike generic-paced breathing, which has demonstrated variable standalone effects on anxiety (Chin et al., 2024). The Swara-UNB model proposes nostril-specific biasing, supporting (Steffen et al., 2021) recommendation for technique-specific protocol in psychotherapy. HRV improvements vary by breathing approach, and therefore justify targeted approaches".

Although several experimental studies report autonomic changes following unilateral nostril breathing, findings are not entirely consistent across protocols. Variability in breathing pace, intervention duration, participant characteristics, and outcome measures complicates direct comparison between studies. Furthermore, much of the available evidence has been obtained in healthy volunteers rather than clinical populations. Consequently, the current evidence supports cautious optimism rather than definitive clinical recommendations.

## 4 Swara-yoga and nostril-specific psychophysiological regulation

### 4.1 Core principles of Swara-yoga and conscious states

Swara-Yoga is the Science of *Prana* (the vital energy) and describes how the manipulation of the breath can be used to regulate, analyse, and harmonize physiological and psychological processes, and ultimately achieve transcendence (Swami, 1997; Shiva Swarodaya (trans.), 1984). *Swara* means the sound and flow of one's own breath, while Yoga means Union; thus, Swara-Yoga represents the union of individual consciousness with supreme consciousness through breath awareness and regulation. While Swara-Yoga is related to *Pranayama* (breath control techniques), it includes aspects beyond mere direction, storage, or retention of prana, including the analysis of pranic rhythms, nostril dominance, and their effects on the functions of the nervous system (Swami, 1997).

Traditionally, Swara-Yoga originated with Lord Shiva (Adinath), who revealed its knowledge to Devi Parvati. This discourse constitutes the foundational text of Swara-Yoga, the *Shiva-Swarodaya*, which proclaims Swara-Yoga as the ‘highest and most secret knowledge' (Shiva Swarodaya (trans.), 1984). Discussions complementary to the Shiva-Swarodaya appear in the Shiva Samhita, Goraksha Samhita, and select Upanishads, establishing Swara-Yoga as part of traditional yogic literature (Gheranda (trans.), 1999).

In this conceptual analysis, as noted earlier, we use *consciousness* in a pragmatic, clinical sense to refer to the organization and quality of awareness—how present, integrated, and responsive an individual is to internal and external experience—rather than to metaphysical debates about the nature of mind. Classical Swara literature proposes that breath rhythms and nostril dominance shape this experiential field. Right-nostril (*Pingala*) dominance is associated with outward-focused, mobilized, task-oriented consciousness; left-nostril (*Ida*) dominance with inward-focused, reflective, calming consciousness; and balanced bilateral flow (*Sushumna*) with more integrated, meditative states in which attention, affect, and bodily experience cohere (; Shiva Swarodaya (trans.), 1984). From this perspective, Swara practice does not merely change physiology; it is a systematic method for moving between qualitatively different states of consciousness.

Contemporary psychophysiology and neuroscience provide converging evidence for a breath–consciousness link compatible with Swara claims. Slow nasal breathing and pranayama practices have been shown to modulate heart rate variability, cardiorespiratory coupling, and large-scale brain networks, resulting in non-ordinary yet organized states of consciousness characterized by altered interoceptive awareness, time perception, and affective tone (Garcia et al., 2013; Pal et al., 2014; Zaccaro et al., 2022). For example, unilateral nostril breathing differentially biases sympathovagal balance (Pal et al., 2014), while slow nasal pranayama practices are associated with changes in cortical oscillations and integrative brain activity in experienced practitioners (Zaccaro et al., 2022). Taken together, these findings suggest that nostril-specific and slow breathing techniques can be understood as natural methods of neuromodulation that shape conscious experience by modulating autonomic and neural dynamics (Bhargav et al., 2026). Within the present Swara-informed model, we therefore interpret Swara-based unilateral nostril breathing as a means of deliberately shifting both the autonomic and conscious states. wara-informed breathing is not an ancillary relaxation tool but a targeted breath–consciousness intervention embedded within psychotherapy.

### 4.2 The three Swaras and nadis

According to Swara-Yoga, there are three primary breath-flows (*Swaras*): the left nostril (*Ida Nadi*), the right nostril (*Pingala Nadi*), and the simultaneous bilateral flow (*Sushumna Nadi*). Observations have established that breathing predominantly occurs through one nostril at a time, with the other partially blocked, alternating approximately every 60–80 min (Shiva Swarodaya (trans.), 1984; Swami, 1997). This alternating rhythm influences physiological and psychological processes, and modulates hemispheric brain activity: *Ida* (left nostril) activates the right hemisphere, *Pingala* (right nostril) activates the left hemisphere, and Sushumna engages whole-brain integration (Shiva Swarodaya (trans.), 1984; Werntz et al., 1987).

Irregularities in this pattern disrupt homeostasis, resulting in psychosomatic dysfunction. Right-Swara is associated with physical/mobilizing activities, left-Swara with mental/reflective activities, and Sushumna with spiritual/transcendent pursuits. To optimize functioning, it is necessary to align activities with the predominant Swara; if activities are mismatched, then inefficiency or distress will result (Swami, 1997).

### 4.3 Recognition and manipulation of Swara

Recognition of Swara involves several easy-to-use techniques. Exhaling onto the palm to compare the strength of each nostril, or comparing the pitch of each exhalation with one nostril occluded (higher pitch indicates closure) (Shiva Swarodaya (trans.), 1984). Equal bilateral flow is indicative of Sushumna, which is considered auspicious for spiritual practices.

Manipulation techniques include: (1) inhaling through the active nostril and exhaling through the inactive one (5–10 min); (2) occluding the active nostril and breathing solely through the desired one; or (3) lying on the side of the active nostril while practicing the aforementioned techniques (Swami, 1997). These allow individuals to align their activities with their physiological rhythms and restore harmony throughout the body, mind, and spirit.

### 4.4 Psychophysiological correlates and modern evidence

Some traditional claims regarding the effects of Swara Yoga have received partial empirical support. Right-nostril dominance has correlated with increased left-hemispheric EEG activity and sympathetic markers, while left-nostril dominance has correlated with right-hemispheric activity and parasympathetic markers, mirroring the descriptions provided in Swara Yoga (Werntz et al., 1987; Block et al., 1989). Pal et al. (2014) reported that right-nostril breathing increased sympathetic HRV markers (LFnu, LF/HF), while left-nostril breathing increased parasympathetic HRV markers (HFnu), consistent with Pingala mobilization and Ida calming. Thus, these results suggest that Swara Yoga may influence autonomic state and therefore provide a preliminary rationale for cautious clinical translation and empirical testing (Pal et al., 2014; Telles et al., 2024).

### 4.5 Distinguishing empirical evidence from the proposed model

Although several studies suggest that unilateral nostril breathing influences autonomic indices such as heart rate variability, the evidence remains limited by small sample sizes, heterogeneous methodologies, and a predominance of healthy participants. Accordingly, the present model should be interpreted as a conceptual framework rather than an evidence-based clinical protocol. The proposed mapping of left nostril breathing to hyperarousal and right nostril breathing to hypoarousal represents a theoretical synthesis derived from Swara Yoga principles and contemporary autonomic research, rather than a clinically established intervention.

## 5 A Swara-inspired autonomic synchrony model for psychotherapy

### 5.1 Rationale: state-based classification

As described earlier, Autonomic Synchrony is conceptualized as the alignment between Swara patterns (nostril-specific breath flow: Ida, Pingala, Sushumna) and autonomic nervous system state, as indexed by heart rate variability (HRV) and related physiological markers (e.g., LF/HF ratio, HF power). When nasal dominance and autonomic indicators move in a coherent, predictable relationship—such as left-nostril (*Ida*) breathing producing parasympathetic-biased HRV changes and right-nostril (*Pingala*) breathing producing sympathetic-biased changes—breath and autonomic physiology function as a coupled system rather than independent processes (Pal et al., 2014; Billman, 2011; Garcia et al., 2013). In the proposed model, Swara-based unilateral nostril breathing is not merely an arousal modulation technique, but a method for deliberately creating breath–autonomic synchrony in the service of psychotherapy process work.

The Swara model elegantly translates to psychotherapy, as the autonomic state determines the accessibility of the process. Instead of relying on rigid diagnoses that may inadequately capture clinical heterogeneity, we recommend a state-based nostril-mapping approach. PTSD encompasses hyperarousal and dissociative subtypes, depression encompasses agitated and psychomotor-retarded presentations, and anxiety disorders include varying arousal levels (Beutler et al., 2022; U.S Department of Veterans Affairs, 2018).

Rather, we propose state-based synchrony grounded in Swara principles and in preliminary psychophysiological research. Therefore, we propose left-nostril (Ida) breathing for hyperarousal states (Sympathetic Dominance), right-nostril (Pingala) breathing for hypoarousal states (Vagal Shutdown), and Sushumna observation (Bilateral Flow) for balanced/spiritual-integration moments (Beutler et al., 2022; Shiva Swarodaya (trans.), 1984; Werntz et al., 1987). In all these cases, the goal is to match the client's current autonomic and experiential state, and to use Swara-informed breath manipulation to bring breath, autonomic activity, and conscious awareness into a configuration that widens the window of tolerance. This approach utilizes the Swara principle of rhythmic analysis and nostril manipulation as adjuncts to evidence-based treatments (Steffen et al., 2021).

This model aligns with the studies on the psychotherapy process, emphasizing momentary state regulation as a transdiagnostic change mechanism, and positioning Swara-UNB as a culturally relevant, low-tech option, testable for additive effects in clinical settings as HRV-optimized psychotherapy adjuncts (Hayes and Hofmann, 2018).

### 5.2 Category a: mobilization / hyperarousal states

**Clinical markers and presentations:** Hyperarousal in clinical terms refers to a state of sympathetic hyperactivity that manifests as physiological mobilization (elevated heart rate, lower heart rate variability), increased vigilance, exaggerated startle response, autonomic surges (palpitations, tremors, etc), heightened irritability/agitation, threat-focused cognition, and sleep onset difficulties. These symptoms are often shown in panic disorder, generalized anxiety disorder, hyperarousal-dominant post-traumatic stress disorder (PTSD), obsessive-compulsive disorder (OCD), urgency states, and mixed/agitated depression (U.S Department of Veterans Affairs, 2013; Beutler et al., 2022).

**Swara prescription:** Breathing through the left nostril (Ida). Slow breathing through the left nostril (Ida) is prescribed due to Ida's parasympathetic down-regulating properties and empirically based parasympathetic biasing (Pal et al., 2014; Shiva Swarodaya (trans.), 1984). 5–10 min of slow left nostril breathing is suggested for clients experiencing hyperarousal states prior to engaging in exposure, cognitive restructuring, or trauma narration. This is hypothesized to reduce the hyperarousal state and potentially expand the window of tolerance (Beutler et al., 2022; Steffen et al., 2021; Craske et al., 2014).

**Integration protocol:** Once swara recognition has been identified using either palm testing or exhalation pitch testing, guide the client to breathe through the left nostril (Ida) for 5–10 min before proceeding with interoceptive exposure. Monitor HRV/subjective arousal levels both before and after the procedure.

**Expected outcome:** Reduced sympathetic hyperarousal will facilitate habituation to anxiety cues without avoidance (Steffen et al., 2021).

### 5.3. Category B: shutdown / hypoarousal states

**Clinical markers and presentations:** Hypoarousal clinically represents dorsal vagal dominance, characterized by reduced sympathetic outflow (reduced behavioral drive), psychomotor retardation, emotional numbing, social withdrawal, anhedonia, and motivational paralysis. These characteristics are representative of the manifestations of melancholic depression, dissociative PTSD subtype (depersonalization/derealization), negative-symptom schizophrenia spectrum, and burnout/exhaustion states (U.S Department of Veterans Affairs, 2018; Beutler et al., 2022; Lanius et al., 2010).

**Swara prescription:** Breathing through the right nostril (Pingala). Right nostril breathing is hypothesized to activate the mobilizing quality of Pingala, and sympathetic biasing, thereby activating sympathetic functions, behavioral activation or grounding (Pal et al., 2014; Shiva Swarodaya (trans.), 1984). Use right nostril breathing for approximately 5-10 min to activate sympathetic functions, preparing for behaviors associated with behavioral activation or grounding.

**Integration protocol:** Guide the client to undergo swara recognition (palm test/exhalation pitch) and then use right nostril breathing to activate sympathetic function in preparation for initiating priming graded activity scheduling (SAS Publishers, 2015).

**Expected outcome:** Counter anhedonic avoidance patterns by increasing the client's sympathetic tone sufficiently to initiate tasks associated with behavioral activation (Dimidjian et al., 2011). Behavioral activation efficacy relies on the client's ability to initiate behaviors. Therefore, it is important to increase the client's sympathetic tone to some extent to facilitate initial task engagement.

### 5.4 Decision rules, sushumna, and clinical flow

Clinical application of Swara-informed breathing procedures will follow a structured four-step protocol utilizing behavioral observations in conjunction with Swara recognition techniques. Initially, clinicians will assess the client's primary autonomic state through behavioral markers (psychomotor agitation vs. retardation, vigilance vs. withdrawal) and Swara recognition (palm test or exhalation pitch comparison) to determine whether the client is primarily exhibiting hyperarousal, hypoarousal, or a balanced state. Next, utilize state-contingent nostril selection. Utilize left nostril breathing (Ida) for hyperarousal states requiring parasympathetic down-regulation, right nostril breathing (Pingala) for hypoarousal states requiring sympathetic mobilization, or Sushumna (bilateral flow) observation to indicate integration moments where relational or reflective work may occur without any therapeutic intervention. Third, after identifying a suitable state for the client, the client will perform unilateral nostril breathing (UNB) for up to 10 min at a comfortable rate (approximately 6 breaths/minute). It is recommended that the client sit in a non-straining position during the procedure and that the procedure be discontinued immediately upon the client's report of discomfort. Finally, assess the client's subjective arousal (SUDS 0-10) or physiological markers (if wearable HRV is available) before progressing to the client's core therapy task, ensuring the client receives state-specific intervention sequencing. This iterative framework supports integrating breathing-based modulation with graded activity scheduling, maintaining alignment with evidence-based interventions while drawing on Swara Yoga's emphasis on rhythmic precision.

**Figure 1:**
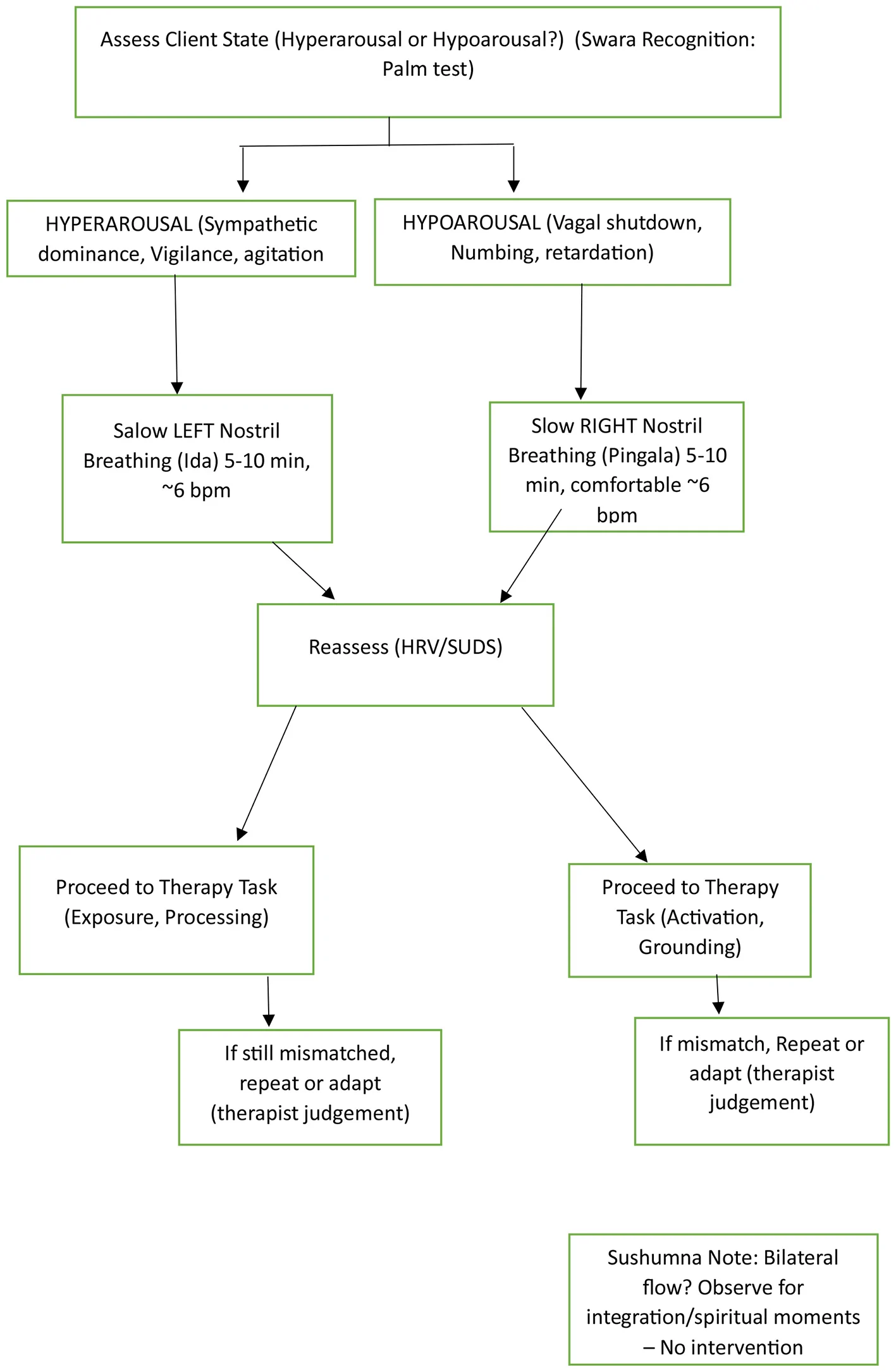
Swara-informed clinical decision protocol for state-based unilateral nostril breathing integration in psychotherapy.

Conceptually, the four-step clinical protocol in Figure 1 operationalises autonomic synchrony as an iterative alignment process. First, therapists assess the client's dominant autonomic and experiential state (hyperarousal, hypoarousal, or balanced) using behavioral markers, subjective reports, and simple Swara recognition. Second, they select the nostril breathing pattern that, based on Swara principles and HRV evidence, should bring breath and autonomic activity into a more functional synchronized configuration for the upcoming therapy task (Pal et al., 2014; SAS Publishers, 2015). Third, brief unilateral nostril breathing is implemented, followed by re-assessment of physiological (e.g., HRV, when available) and subjective arousal indices.

## 6 Integration with psychotherapy practice

### 6.1 Matching with CBT, trauma-focused work, and other modalities

Unilateral nostril breathing (UNB) may function best as a short-term, state-responsive adjunct to existing psychotherapeutic protocols. Therefore, UNB enhances access to the core mechanisms of psychotherapy without replacing them (Pal et al., 2014; Steffen et al., 2021). In CBT for anxiety disorders, Swara recognition followed by 5–10 min of slow left nostril breathing can be used to prepare the client for interoceptive exposure or cognitive restructuring prior to the actual therapy session, thereby lowering the client's sympathetic tone so that they can better tolerate anxiety cues while maintaining the hierarchical structure of the exposure (Beutler et al., 2022; Pal et al., 2014). Similarly, in CBT for depression presenting as a hypoarousal-dominant state, right nostril breathing can be used as a mobilization primer just prior to behavioral activation scheduling to assist the client in initiating graded activities without becoming overly agitated (SAS Publishers, 2015). Trauma-focused therapies, such as prolonged exposure (PE) or eye movement desensitization and reprocessing (EMDR), also benefit from Swara-informed breathing procedures. Left nostril breathing aligns with Ida's parasympathetic down-regulating properties to stabilize hyperarousal in the client prior to imaginal exposure or bilateral stimulation, thereby widening the client's window of tolerance for trauma narrative processing (Shiva Swarodaya (trans.), 1984; U.S Department of Veterans Affairs, 2013). For dissociative PTSD presentations, right nostril breathing can assist the client to slowly counter shutdown states before engaging in grounding exercises or resourcing phases, thereby assisting the client in safely re-engaging in memory work (U.S Department of Veterans Affairs, 2018). Dialectical behavior therapy (DBT) skills training may incorporate Swara-informed nostril selection as a distress tolerance skill, matched to the client's present momentary biosocial states (Beutler et al., 2022).

Regardless of the modality, UNB is strictly adjunctive to the client's evidence-based treatment plan. UNB assists in preparing the client's autonomic substrate for the evidence-based mechanisms of the client's treatment plan (exposure, cognitive reappraisal, relational repair), but does not constitute the treatment itself, thereby preserving empirical integrity while leveraging cultural resonance (Steffen et al., 2021).

### 6.2 Practical implementation of the Swara-informed intervention

In practical terms, the Swara-informed intervention is intended to function as a brief, structured pre-task regulation procedure embedded within an ongoing course of evidence-based psychotherapy rather than as an independent treatment modality. Its proposed purpose is to improve the client's readiness for therapeutic work by shifting momentary autonomic state toward a more workable zone of arousal before exposure, trauma processing, cognitive restructuring, grounding, or behavioral activation. In this framework, unilateral nostril breathing (UNB) is used to prepare the psychophysiological conditions under which established therapeutic techniques may be more tolerable and effective, while the primary mechanisms of change remain those of the host treatment itself.

Implementation may follow a simple five-step sequence. First, the therapist conducts a brief state assessment at the start of the relevant session segment, using observable behavioral and affective markers such as agitation, hypervigilance, autonomic surge, emotional flooding, psychomotor slowing, withdrawal, numbing, or dissociative disengagement to determine whether the client presents predominantly in a hyperarousal or hypoarousal state. Second, the therapist explains the exercise in clear, non-mystifying language, obtains informed consent, and frames the practice as an exploratory, adjunctive self-regulation tool rather than an evidence-based treatment in its own right. Third, a brief Swara check may be performed using simple nostril-awareness methods already described above, although the intervention may also be administered on the basis of clinical state assessment when formal nostril testing is impractical.

Fourth, the client engages in slow unilateral nostril breathing in a comfortable seated posture without breath retention, strain, or forced inhalation. For hyperarousal-dominant states, slow left nostril breathing is proposed as a down-regulating adjunct before tasks such as interoceptive exposure, trauma narration, emotionally intense cognitive work, or distress-tolerance practice. For hypoarousal-dominant states, slow right nostril breathing is proposed as a gentle mobilization strategy before grounding work, behavioral activation, or task initiation. In the current conceptual model, a conservative implementation window of approximately 2–5 min may be used initially, with extension up to 5–10 min when the client tolerates the procedure well and the clinical context permits. Fifth, the therapist briefly reassesses subjective arousal, behavioral readiness, and, where feasible, physiological markers such as HRV before proceeding to the planned psychotherapy task.

This procedure is best understood as a session-level preparatory intervention rather than a stand-alone daily prescription. It may be particularly relevant at identifiable transition points in therapy, such as immediately before exposure exercises, trauma-focused processing, behavioral activation assignments, or re-engagement following shutdown or flooding during the session. If the client becomes more dysregulated, uncomfortable, dizzy, agitated, or emotionally detached during the exercise, the breathing practice should be stopped, and the clinician should return to ordinary grounding, pacing, or other established regulation strategies. Accordingly, the present model supports only cautious, therapist-monitored, and explicitly adjunctive use, pending stronger empirical evidence regarding efficacy, safety, therapist effects, and long-term outcomes.

### 6.3 Cultural and ethical considerations

The growing body of research on yoga has led to the global acceptance of yoga as a mainstream complementary therapy for a variety of psychophysiological conditions, including anxiety, depression, PTSD, and chronic stress (Cramer et al., 2018a,b; World Health Organization, 2021). Pranayama, or nostril breathing (unilateral nostril breathing), has shown moderate to large effect sizes on heart rate variability (HRV), cortisol, and symptom reductions across multiple systematic reviews, demonstrating their utility as adjunct treatment strategies in various clinical settings (Sivaramakrishnan et al., 2019; Morgan et al., 2024). The swara-informed model builds on this established infrastructure and may have potential applicability across Western psychotherapy clinics, Indian mental health centers, community yoga programs, and global telemedicine platforms.

In India, Swara Yoga continues to have great cultural relevance through thousands of years of yoga tradition and practice and, consequently, enjoys greater acceptability, adherence, and meaningfulness among clients with Hindu, Tantric, and yogic backgrounds (Swami, 1997; Shiva Swarodaya (trans.), 1984). Global adaptation reconceptualises unilateral nostril breathing (UNB) as a culturally responsive, evidence-based method of autonomic regulation, potentially introducing Ida/Pingala terminology for interested clients, while reinforcing universally applicable principles (i.e. sympathetic/parasympathetic balance) (Werntz et al., 1987; Pal et al., 2014).

Consistent with standards of care and professional ethics, informed consent will require that the client receive full disclosure of expected effects (i.e. state-specific arousal modulation), the current level of evidence supporting these effects (e.g. emerging HRV-related studies and their role as an adjunct intervention), possible discomforts (e.g. dizziness or temporary increases in anxiety), and the voluntary nature of client participation (American Psychological Association, 2017). Consistent with cultural humility, clinicians will need to continuously assess fit with the client's values, beliefs, etc. (modifications for low resonance, discontinuation if not suitable) to ensure ethical use of this technique across diverse cultural environments (Hook et al., 2017).

This model's setting-agnostic design (5–10-min protocols, minimal training) supports implementation across contexts ranging from resource-rich academic centers to community-based interventions worldwide, leveraging yoga's established global infrastructure.

### 6.4 Safety and precautions

UNB is considered low risk when administered cautiously. However, there are precautions to consider. Clients who exhibit hyperarousal sensitive responses (e.g. panic disorder, high interoceptive anxiety) may experience paradoxical escalation. Therefore, conduct 2-min trials, monitor the client's subjective units of distress (SUDs) levels, and cease the trial if the client reports SUDs greater than 7/10 (Pal et al., 2014). Cardiovascular instability, a recent myocardial infarction, or uncontrolled hypertension will preclude the use of UNB until medical clearance is obtained, due to varying blood pressure responses (SAS Publishers, 2015). Clients with manic, hypomanic, or mixed bipolar states should refrain from using right nostril breathing due to mobilization risk (Beutler et al., 2022).

#### 6.4.1 Recommended protocol safeguards:

- Bouts lasting no longer than 10 min; non-stressing seated position.
- The practice does not require breath retention, forcing, or strain.
- Stopping criteria: discontinue the intervention if participants experience dizziness, headache, panic escalation, or discomfort exceeding a moderate level.
- Return to normal breathing and reassess the client's suitability to continue the intervention.

With respect to supervision, trained clinicians will ensure the client's safety and document client progress via session notes and client feedback (Steffen et al., 2021).

## 7 Testable hypothesis and research agenda of swara-informed autonomic synchrony model

The Swara-informed autonomic matching model is intended to be both empirically testable and falsifiable, rather than accepted as a clinical fact based on tradition. The central claim is that unilateral nostril breathing (UNB) adds *state-contingent specificity* to paced-breathing approaches already discussed in contemporary psychotherapy integration work.

### 7.1 Specific, falsifiable hypotheses

Each hypothesis shall be stated to reduce ambiguity using the (i) population/state, (ii) timing, (iii) comparator, (iv) measurable endpoint(s), and (v) predicted direction.

**H1** (Hyperarousal down-regulation; process). Among clients presenting with a hyperarousal-dominant state (e.g., hypervigilant PTSD, panic-focused anxiety), brief slow left nostril breathing immediately before exposure-based or trauma-focused procedures will produce a *greater parasympathetic shift*, operationalised as increased HRV indices commonly interpreted as vagal activity (e.g., HFnu) and reduced indices interpreted as sympathetic dominance (e.g., LF/HF ratio), and a greater reduction in subjective arousal state (SUDS) compared with neutral paced breathing matched for rate and duration.

**H2** (Hypoarousal mobilization; process + behavior)

Among clients presenting in a hypoarousal-dominant state (shutdown/numbing/psychomotor slowing; including dissociative PTSD presentations), brief slow right nostril breathing immediately before behavioral activation or grounding procedures will produce a *mobilization shift*, operationalised as changes in HRV frequency-domain indices consistent with the right-nostril pattern reported in UNB trials (e.g., increased LFnu with decreased HFnu) plus increased “readiness-to-act” ratings, and will increase initiation of the planned therapy task (e.g., starting an assigned micro-activity within-session; completing first-step homework within 24 h) compared with neutral paced breathing matched for rate and duration.

**H3** (State-matching superiority; comparative effectiveness).

Across a heterogeneous clinical sample, state-matched UNB (left for hyperarousal; right for hypoarousal) will outperform state-mismatched UNB and neutral paced breathing on (a) session-by-session arousal regulation (physiological + subjective) and (b) treatment engagement (attendance, homework completion, reduced avoidance/safety behaviors).

**H4** (Mechanistic mediation; process-specificity).

The clinical benefits of state-matched UNB (symptom reduction; improvement in functioning) will be mediated by within-session improvements in arousal regulation (HRV change, SUDS stabilization), rather than non-specific factors such as relaxation, expectancy, or therapist attention.

**H5** (Cultural moderation; implementation).

In contexts where yoga and Indian knowledge systems are familiar, Swara-framed delivery (Ida/Pingala/Sushumna language offered with consent) will be more acceptable and adhered to than a purely neutral biomedical framing. These implementation gains will partially explain improved engagement outcomes.

**Built-in falsifiability (recommended to state explicitly)**.

If (i) left vs right nostril breathing does not differ from neutral paced breathing when rate/dose are controlled, and (ii) state-matched assignment does not outperform mismatched assignment, then the nostril-specific component of the model is not supported, and Swara-UNB should be treated as a generic paced-breathing adjunct rather than a specific synchrony method.

### 7.2 Study designs that directly test the model

**Design A:** Mechanism-focused, within-session RCT (fastest path to evidence).

Randomize sessions (or session blocks) to: (1) state-matched UNB, (2) state-mismatched UNB, (3) neutral paced breathing (rate-matched), keeping the psychotherapy task constant (e.g., interoceptive exposure, imaginal exposure, or behavioral activation). This design isolates whether nostril selection adds value beyond paced breathing, a key methodological issue raised in reviews of breathing-based interventions.

**Design B:** Adjunct RCT across diagnoses (effectiveness + generalisability).

Incorporate UNB as a standardized 5-10 min pre-task module across manualised interventions (CBT for GAD/panic; exposure-based PTSD work; behavioral activation for depression) and stratify by dominant state at session start. Primary outcomes can include symptom scales and functioning, with process outcomes (HRV/SUDS) measured session-by-session to test H4 mediation.

A pragmatic randomized controlled trial will test UNB as a standardized 5–10 min pre-task module across manualised interventions: CBT for GAD/panic disorder, exposure therapy for PTSD, and behavioral activation for depression. Participants will be stratified by dominant autonomic state (hyperarousal vs. hypoarousal) at session onset. Primary outcomes are symptom severity and functioning. Process measures (HRV during UNB-to-task transition; SUDS during exposure/activation) will test H4 mediation via multilevel modeling.

**Design C:** Micro-randomized or adaptive design (matches our “state-based” claim).

Use repeated, brief randomisations within and across sessions, where assignment depends on measured state (hyper vs hypo), to test whether dynamic state-matching improves momentary regulation and downstream engagement. This approach fits the core premise that the state fluctuates and that matching should be responsive rather than diagnosis-fixed.

### 7.3 Measurement strategy (minimal but adequate)

- **Physiology (process):** Wearable ECG-derived HRV; report time-domain (e.g., RMSSD) and frequency-domain indices used in UNB trials (HFnu, LFnu, LF/HF), with standardized artifact handling and a fixed measurement window pre/post UNB and during the therapy task.
- **Subjective state (process):** SUDS for arousal, and a brief shutdown/dissociation rating for hypoarousal presentations (especially when testing dissociative PTSD).
- **Engagement (intermediate outcome):** Homework completion, attendance, task initiation latency, therapist-rated avoidance/safety behavior occurrence during exposure/activation.
- **Clinical outcomes:** Diagnosis-appropriate symptom severity scales and functioning measures (pre/post treatment), with process endpoints (immediate UNB/SUDS changes) explicitly distinguished from distal outcome endpoints (clinical changes across weeks).

#### 7.3.1 . Addressing Evidence Limitations in Breathing Interventions

Recent systematic reviews highlight the mixed efficacy of standalone breathing interventions for anxiety and other disorders, emphasizing the need for mechanistic refinement (Chin et al., 2024; Morgan et al., 2024). Chin et al. (2024) synthesized 12 RCTs on brief respiratory interventions and found that embodiment and combination practices consistently reduced state anxiety. In contrast, isolated breathing techniques produced inconsistent results (ranging from a significant positive effect to a null effect), and outcomes varied depending on whether the comparison group received an active intervention or merely passive attention. Similarly, Morgan et al. (2024) reported significant reductions in anxiety across 19 breathing studies (p < 0.05), but emphasized the limited number of large RCTs and called for further applications.

The present Swara-UNB model positions nostril-specific breathing as a candidate mechanism that requires demonstration of an *additive benefit* over generic paced breathing, rather than a standalone cure. Unlike generic diaphragmatic breathing, Swara proposes a differential autonomic biasing testable via state-matching designs. This addresses review gaps by hypothesizing that nostril effects enhance existing psychotherapy rather than replace it, with falsification via mismatched conditions showing null superiority (Chin et al., 2024; Steffen et al., 2021).

### 7.4 . Clinical implications

The present framework suggests that UNB may be considered as an adjunctive practice rather than a standalone therapeutic intervention. It should not be viewed as a replacement for evidence-based treatments such as CBT, EMDR, ACT, DBT, or other trauma-focused therapies. Instead, its potential lies in supporting therapeutic readiness and complementing established clinical approaches.

In practice, UNB may be cautiously introduced as a preparatory strategy before emotionally demanding or regulation-focused therapeutic tasks. Its use should always be accompanied by informed consent, with clients clearly informed that the approach remains exploratory and is not yet supported by a strong clinical evidence base. At this stage, any clinical application should therefore be undertaken with caution and framed as hypothesis-informed rather than evidence-based.

For research-oriented or integrative settings, UNB may offer a possible avenue for enhancing patient engagement, grounding, or autonomic preparation prior to psychotherapy. However, stronger empirical evidence is needed before broader clinical recommendations can be made. Until then, its role is best understood as supportive, limited, and provisional.

### 7.5 . Limitations to state up-front (and how the agenda addresses them)

UNB findings are protocol-dependent (pace, dose, state), and “LF/HF as sympathovagal balance” has interpretive limits. Therefore, claims should be framed as *biasing* rather than deterministic switching. Systematic reviews of brief respiratory interventions show variable effects and emphasize the need for better controls and technique-specific dismantling, which is precisely why the matched vs. mismatched vs. neutral paced breathing comparisons are central here. Much of the existing evidence in this area comes from studies involving healthy participants rather than clinical populations, which may limit the generalizability of the present findings to individuals with psychological or medical conditions.

The long-term effects of Swara-based or related interventions are still unknown, and the present study cannot establish the durability of outcomes over time. Finally, because paced breathing alone can produce meaningful autonomic shifts, the research program must demonstrate the incremental validity of nostril-specific matching beyond rate-matched breathing. Otherwise, the Swara component becomes a cultural framing rather than a distinct mechanism.

## 8 Conclusion

The Swara-informed autonomic synchrony model offers a novel, culturally grounded framework for integrating unilateral nostril breathing into psychotherapy practice to address a core clinical challenge: state-contingent autonomic regulation. By associating left nostril (Ida) breathing with hyperarousal states and right nostril (Pingala) breathing with hypoarousal states as brief adjuncts to evidence-based therapies, the model leverages preliminary psychophysiological evidence while honoring the emphasis of Swara Yoga on breath rhythm analysis and harmony. The proposed Swara-informed autonomic synchrony model should be regarded as a conceptual framework intended to stimulate empirical investigation rather than a validated clinical intervention. Future randomized controlled trials are required to determine whether state-matched unilateral nostril breathing provides incremental benefits beyond established paced-breathing techniques.

Viewed through the lens of the Breathing and Consciousness framework, the Swara-informed autonomic synchrony model is not only an arousal-regulation proposal, but also a breath–consciousness model: by matching Swara patterns to autonomic state, the therapist aims to co-regulate physiological rhythms and the client's momentary state of consciousness in ways that make exposure, behavioral activation, and relational repair phenomenologically possible.

This model may be particularly useful in clinical and health care settings where easily accessible, cost-effective regulators can promote increased access to exposure, behavioral activation, and trauma processing. The five testable hypotheses, covering process (HRV), outcome (symptoms), and cultural aspects, provide a clear roadmap for empirical assessment, allowing distinction of nostril-specific benefits from paced-breathing confounds.

While there are potential limitations, including the need for diverse evidence and clinical trials, this model invites rigorous investigation to assess its incremental utility. Successful validation could add a new, mechanistically informed, traditionally rooted intervention to the psychotherapy that integrates traditional wisdom and modern science to improve client outcomes.

## References

[B1] American Psychological Association, (2017). Ethical principles of psychologists and code of conduct. Available online at: https://www.apa.org/ethics/code/ (Accessed March 28, 2026).

[B2] Berntson G. G. Bigger J. T. Jr Eckberg, D. L. Grossman P. Kaufmann P. G. Malik M. Nagaraja H. N. Porges S. W. Saul J. P. Stone P. H. van der Molen M. W. (1997). Heart rate variability: origins, methods, and interpretive caveats. Psychophysiology, 34, 623–648. doi: 10.1111/j.1469-8986.1997.tb02140.x 9401419

[B3] Beutler S. Heeke C. Schlarb A. A. Knaevelsrud C. (2022). Trauma-related dissociation and the autonomic nervous system. Eur. J. Psychotraumatol. 13:2119909. doi: 10.1080/20008066.2022.2132599 PMC9635467 36340007

[B4] Bhargav H. Mehta U. M. Kumar A. Subramanian S. Keshavan M. S. (2026). Breathing and the brain: pranayama, an ancient self-directed approach to neuromodulation. Asian J. Psychiatr. 115:104788. doi: 10.1016/j.ajp.2025.104788 41317714

[B5] Billman G. E. (2011). Heart rate variability: a historical perspective. Front. Physiol. 2:86. doi: 10.3389/fphys.2011.00086 22144961 PMC3225923

[B6] Block R. A. Arnott D. P. Quigley B. Lynch W. C. (1989). Unilateral nostril breathing influences lateralized cognitive performance. Brain Cogn. 9, 181–190. 2923709 10.1016/0278-2626(89)90028-6

[B7] Chin P. Lomas J. Ivtzan I. (2024). A systematic review of brief respiratory, embodiment, cognitive, and mindfulness interventions to reduce state anxiety. Front. Psychol. 15:1412928. doi: 10.1016/0278-2626(89)90028-6 38933581 PMC11203600

[B8] Cramer H. Lauche R. Anheyer D. Pilkington K. de Manincor M. Dobos G. . (2018a). Yogic breathing meta-analysis. 10.1002/da.22762 29697885

[B9] Cramer H. Lauche R. Langhorst J. Dobos G. (2018b). Yoga for depression: a systematic review and meta-analysis. Depress. Anxiety 35, 830–843. doi: 10.1002/da.22762 23922209

[B10] Craske M. G. Treanor M. Conway C. C. Zbozinek T. Vervliet B. (2014). Maximizing exposure therapy: an inhibitory learning approach. Behav. Res. Ther. 58, 10–23. doi: 10.1016/j.brat.2014.04.006 24864005 PMC4114726

[B11] Dimidjian S. Barrera M. Jr Martell C. Muñoz R. F. Lewinsohn P. M. (2011). The origins and current status of behavioral activation treatments for depression. Annu. Rev. Clin. Psychol. 7, 1–38. doi: 10.1146/annurev-clinpsy-032210-104535 21275642

[B12] Foa E. B. Hembree E. A. Cahill S. P. Rauch S. A. M. Riggs D. S. Feeny N. C. . (2005). Randomized trial of prolonged exposure for posttraumatic stress disorder with and without cognitive restructuring: outcome at academic and community clinics. J. Consult. Clin. Psychol. 73, 953–964.doi: 10.1037/0022-006X.73.5.953 16287395

[B13] Garcia A. J. III. Koschnitzky J. E. Dashevskiy T. Ramirez J.-M. (2013). Cardiorespiratory coupling in health and disease. Auton. Neurosci. 175, 26–37. doi: 10.1016/j.autneu.2013.02.006 23497744 PMC3683976

[B14] Geisler F. C. M. Vennewald N. Kubiak T. Weber H. (2010). The impact of heart rate variability on subjective well-being is mediated by emotion regulation. Pers. Individ. Dif. 49, 723–728. doi: 10.1016/j.paid.2010.06.015

[B15] Gerritsen R. J. S. Band G. P. H. (2018). Breath of life: the respiratory vagal stimulation model of contemplative activity. Front. Hum. Neurosci. 12:397. doi: 10.3389/fnhum.2018.00397 30356789 PMC6189422

[B16] Gheranda S. (1999). Gheranda Samhita (Transl.by Swami Niranjanananda,). Munger: Bihar School of Yoga.

[B17] Hayes S. C. Hofmann S. G. eds. (2018). Process-based CBT: The Science And Core Clinical Competencies of Cognitive Behavioral Therapy. Oakland, CA: New Harbinger Publications.

[B18] Hofmann S. G. Smits J. A. J. (2008). Cognitive-behavioral therapy for adult anxiety disorders: A meta-analysis of randomized placebo-controlled trials. J. Clin. Psychiatry 69, 621–632. doi: 10.4088/jcp.v69n0415 18363421 PMC2409267

[B19] Hook J. N. Davis D. E. Owen J. Worthington E. L. Utsey S. O. (2017). Cultural humility: measuring openness to culturally diverse clients. J. Couns. Psychol. 64, 353–366. doi: 10.1037/a0032595 23647387

[B20] Lanius R. A. Vermetten E. Loewenstein R. J. Brand B. Schmahl C. Bremner J. D. Spiegel D. (2010). Emotion modulation in PTSD: Clinical and neurobiological evidence for a dissociative subtype. Am. J. Psychiatry 167, 640–647. doi: 10.1176/appi.ajp.2009.09081168 20360318 PMC3226703

[B21] Morgan S. P. Lengacher C. A. Seo Y. (2024). A systematic review of breathing exercise interventions: an integrative complementary approach for anxiety and stress in adult populations. Biol. Res. Nurs. 43, 354–376. doi: 10.1177/08980101241273860 39150318

[B22] Muktibodhananda S. (1984/2006). Swara Yoga: The Tantric Science of Brain Breathing (Rev. ed.). Munger: Yoga Publications Trust.

[B23] Ogden P. Minton K. Pain C. (2006). Trauma and the Body: A Sensorimotor Approach to Psychotherapy. New York, NY: W. W. Norton & Company.

[B24] Pal G. K. Agarwal A. Karthik S. Pal P. Nanda N. (2014). Slow yogic breathing through right and left nostril influences sympathovagal balance, heart rate variability, and cardiovascular risks in young adults. N. Am. J. Med. Sci. 6, 145–151. doi: 10.4103/1947-2714.128477 24741554 PMC3978938

[B25] Pozzato I. Haghighi M. Close J. Pin M. Rogers K. (2025). The effects of paced breathing on psychological distress vulnerability and heart rate variability in adults sustaining traumatic injury. J. Affect. Disord. 338, 102–112. doi: 10.1016/j.jad,.2025.01.008 39778745

[B26] SAS Publishers, (2015). Effect of unilateral nostril breathing on autonomic functions in young adults. Int. J. Med. Res. Prof. 1, 90–94.

[B27] Shiva Swarodaya (trans.), (1984). In Muktibodhananda S., Swara yoga: The tantric science of brain breathing.

[B28] Siegel D. J. (1999). The Developing Mind: Toward a Neurobiology of Interpersonal Experience. New York, NY: Guilford Press.

[B29] Sivaramakrishnan D. Fitzsimons C. Kelly P. Ludwig K. Mutrie N. Saunders D. H. . (2019). The effects of yoga compared to active and inactive controls on physical function and health related quality of life in older adults. BMC Geriatr. 19:165. doi: 10.1186/s12966-019-0789-2 30953508 PMC6451238

[B30] Steffen P. R. Gaskins S. Hinderliter A. Sherwood A. (2021). Integrating breathing techniques into psychotherapy to improve HRV: which approach is best? Front. Psychol. 12:624254. doi: 10.3389/fpsyg.2021.624254 33658964 PMC7917055

[B31] Swami M. (1997). Shiva Swarodaya. Varanasi: Chaukhamba Sanskrit Pratishthan.

[B32] Task Force of the European Society of Cardiology and the North American Society of Pacing and Electrophysiology (1996). Heart rate variability: standards of measurement, physiological interpretation, and clinical use. Circulation 93, 1043–1065. doi: 10.1161/01.CIR.93.5.1043 8598068

[B33] Telles S. Yadav A. Kumar N. Sharma S. Visweswaraiah N. K. Balkrishna A. (2013). Blood pressure and Purdue pegboard scores in individuals with hypertension after alternate nostril breathing, breath awareness, and no intervention. Med. Sci. Monit. 19, 61–66. doi: 10.12659/MSM.883743 23334063 PMC3628802

[B34] Telles S. Yadav A. Sharma Y. Balkrishna A. (2024). Heart rate variability during nostril-regulated yoga breathing practices: a randomized trial. Int. J. Yoga 17, 3–12. doi: 10.4103/ijoy.ijoy,_119_24 39959514 PMC11823553

[B35] U.S Department of Veterans Affairs, (2013). Posttraumatic Accessed d date disorder (PTSD) and DSM-5. PTSD: National Center for PTSD. Available online at: https://www.ptsd.va.gov/professional/treat/essentials/dsm5_ptsd.asp.

[B36] U.S Department of Veterans Affairs, (2018). Dissociative subtype of PTSD. PTSD: National Center for PTSD. Available online at: https://www.ptsd.va.gov/professional/treat/essentials/dissociative_subtype.asp

[B37] Vanutelli M. E. Grigis C. Lucchiari C. (2024). Breathing right… or left! The effects of unilateral nostril breathing on psychological and cognitive wellbeing: a pilot study. Brain Sci. 14:302. doi: 10.3390/brainsci14040302 38671954 PMC11048276

[B38] Werntz D. A. Bickford R. G. Shannahoff-Khalsa D. (1987). Selective hemispheric stimulation by unilateral forced nostril breathing. Hum. Neurobiol. 6, 165–171. 3449485

[B40] World Health Organization, (2021). Traditional Medicine Global Report. Geneva: World Health Organization.

[B41] Yadav R. K. Das S. Ghosh S. (2016). A pilot study to check effects of alternate nostril breathing on heart rate variability in healthy volunteers. Indian J. Clin. Exp. Physiol. 3, 526–531.

[B42] Zaccaro A. Piarulli A. Melosini L. Menicucci D. Gemignani A. (2022). Neural correlates of non-ordinary states of consciousness in pranayama practitioners: the role of slow nasal breathing. Front. Syst. Neurosci. 16:803904. doi: 10.3389/fnsys.2022.803904 35387390 PMC8977447

[B43] Zhao X. Li J. Liu Y. Wang Y. Zhang H. (2025). Individualised paced deep breathing training with autonomic targets in chronic heart failure: a randomised controlled trial. Front. Physiol. 16:1458321. doi: 10.3389/fphys.2025.1458321

